# Variation in mutational (co)variances

**DOI:** 10.1093/g3journal/jkac335

**Published:** 2022-12-22

**Authors:** François Mallard, Luke Noble, Charles F Baer, Henrique Teotónio

**Affiliations:** Institut de Biologie de l’École Normale Supérieure, PSL Research University, CNRS UMR 8197, Inserm U1024, F-75005 Paris, France; Institut de Biologie de l’École Normale Supérieure, PSL Research University, CNRS UMR 8197, Inserm U1024, F-75005 Paris, France; Department of Biology, University of Florida Genetics Institute, University of Florida, Gainsville, FL 32611, USA; Institut de Biologie de l’École Normale Supérieure, PSL Research University, CNRS UMR 8197, Inserm U1024, F-75005 Paris, France

**Keywords:** **M**-matrix, **G**-matrix, multivariate selection, locomotion behavior, transition rates, experimental evolution, *Caenorhabditis elegans*

## Abstract

Because of pleiotropy, mutations affect the expression and inheritance of multiple traits and, together with selection, are expected to shape standing genetic covariances between traits and eventual phenotypic divergence between populations. It is therefore important to find if the **M** matrix, describing mutational variances of each trait and covariances between traits, varies between genotypes. We here estimate the **M** matrix for six locomotion behavior traits in lines of two genotypes of the nematode *Caenorhabditis elegans* that accumulated mutations in a nearly neutral manner for 250 generations. We find significant mutational variance along at least one phenotypic dimension of the **M** matrices, but neither their size nor their orientation had detectable differences between genotypes. The number of generations of mutation accumulation, or the number of MA lines measured, was likely insufficient to sample enough mutations and detect potentially small differences between the two **M** matrices. We then tested if the **M** matrices were similar to one **G** matrix describing the standing genetic (co)variances of a population derived by the hybridization of several genotypes, including the two measured for **M**, and domesticated to a lab-defined environment for 140 generations. We found that the **M** and **G** were different because the genetic covariances caused by mutational pleiotropy in the two genotypes are smaller than those caused by linkage disequilibrium in the lab population. We further show that **M** matrices differed in their alignment with the lab population **G** matrix. If generalized to other founder genotypes of the lab population, these observations indicate that selection does not shape the evolution of the **M** matrix for locomotion behavior in the short-term of a few tens to hundreds of generations and suggests that the hybridization of *C. elegans* genotypes allows selection on new phenotypic dimensions of locomotion behavior.

## Introduction

The **G** matrix, the additive genetic (co)variance matrix summarizing how multiple traits are genetically structured and inherited from parents to offspring, provides both prospective and retrospective information about phenotypic evolution. Looking forward, the evolution of mean trait values over one generation, $\Delta \bar{z}$, can be predicted from Lande’s equation (Lande 1979): $\Delta \bar{z}=G\beta$, where β is the vector of directional selection gradients (Lande 1976; Lande and Arnold 1983). Similarly, using the multivariate version of Bulmer’s equation, the evolution of trait variances depends on **G** and linkage disequilibrium (LD) generated by past selection (Bulmer 1971; Tallis 1987). Looking backward, the net selection gradients responsible for mean multivariate trait divergence between populations over multiple generations can be estimated from $\beta =G^{-1}({\bar{z}}_{a}-{\bar{z}}_{b})$ (Lande 1979), where ${\bar{z}}_{a}$ and ${\bar{z}}_{b}$ are the vectors of trait means for the ancestral population *a* and the derived evolved population *b*. In this later case, the accuracy and precision of the inferences made depends on the stability of **G** over multiple generations (Shaw *et al.* 1995; Schluter 1996; Arnold *et al.* 2001). However, and even in the absence of selection, **G** cannot be stable in the short-term of tens to hundreds of generations, as mutation and genetic drift will change its orientation in unpredictable ways (Phillips *et al.* 2001; Barton and Turelli 2004; Phillips and McGuigan 2006; Mallard *et al.* 2022).

In the absence of selection, and assuming an infinitesimal model of trait inheritance, drift predictably removes genetic (co)variance from a diploid population at rate (1-$1/2N_{e}$) per generation (Lande 1976; Lynch and Hill 1986; Barton *et al.* 2017), where $N_{e}$ is the effective population size. Mutation introduces genetic (co)variance into the population at rate **M** per generation, where the diagonal elements of **M** are the mutational variances, and the off-diagonals the mutational covariances between traits. In the long-term of mutation-drift equilibrium, $E[G]=2N_{e}M$, and the asymptotic rate of divergence in **G** between populations is 2**M** per generation (Lande 1979; Lynch and Hill 1986; Hansen and Martins 1996; Felsenstein 1988).

Before reaching mutation-drift equilibrium, however, there is no stochastic theory to describe the expected distribution of **G**. One cannot predict the stability of **G** because all but the simplest deterministic models depend on the distribution of mutational effects, which for most traits is likely not normal, e.g. Hayes and Goddard (2001), and third and higher order moments of the distribution might affect the evolution of **G** (Barton and Turelli 1987, 1989; Johnson and Barton 2005).

Examination of a simple deterministic model is nonetheless instructive as it suggests a way to better understand the evolution of **G**. Assuming that mutational effects are multivariate Gaussian and that selection is weak relative to recombination then (Lande 1980):


(1) $$\Delta G=G(\gamma -\beta \beta^{T})G+2M-2\sum_{i=1}^{n}\sum_{\;j=1}^{n}r_{ij}(C_{ij}-C_{ij}^{\prime }),$$

that is, changes in **G** depend on directional selection (β), stabilizing/disruptive selection on single traits and correlated selection between pairs of traits [diagonal and off-diagonal of γ, cf. Lande and Arnold (1983)], together with mutational input and output by recombination. The last term, represents the breakdown of genetic covariance resulting from LD between loci i and j with recombination rate $r_{ij}$; with $C_{ij}$ and $C_{ij}^{\prime }$ being the (co)variances resulting from associations between alleles on the same and different gametes, respectively. In the absence of other factors, selection will cause **G** to evolve to its expected value at a local point where fitness is maximized (Lande 1979; Cheverud 1984; Barton and Turelli 1987; Jones *et al.* 2003). Mutation can cause a buildup of LD in finite populations, but there is no reason to expect that allelic effects of new mutations at different loci are correlated unless mutation rates are high and selection is also important (Hill and Robertson 1966). Under the infinitesimal and infinite population size assumptions implicit in equation (1) (Barton *et al.* 2017), mutational covariance reflects the underlying pleiotropic effects of new mutant alleles. Selection maintains LD between combinations of alleles at different loci (Bulmer 1976; Turelli 1988), in which case genetic covariance results from both LD and pleiotropy. However, LD should be rapidly dissipated by recombination unless loci are very tightly linked or assortative mating is strong (Lande 1980). As a consequence, ΔG≈0 when the input of new genetic (co)variance by mutation offsets the (co)variance created by selection.

The deterministic model in equation (1) suggests that, when a balance between mutation and selection is reached, then the orientation of **G** will match that of **M**, and by extension, the orientation of the phenotypic divergence among taxa (**D**) will match that of **M** (Lande 1979). Two studies have found evidence for such congruence between **M** and **G** in *Drosophila* species wing shape (Houle *et al.* 2017; Dugand *et al.* 2021). Two other studies have found evidence for congruence between the orientations of **M** and **D**, one encompassing 40 million years of Drosophilid fly wing shape evolution (Houle *et al.* 2017), another encompassing 100 million years of Rhabditid nematode embryo size evolution (Farhadifar *et al.* 2015). Many other studies have further shown positive correlations between mutation and additive genetic variances (the diagonal elements of **M** and **G**) consistent with mutation–selection balance predictions, reviewed in Farhadifar *et al.* (2016). These results are remarkable because **M** was in some studies measured in different genotypes but effectively encompassing only a single “averaged” genetic background of a single population from a single species; see Discussion. How can genetic and phenotypic evolution be predictable in the very long term of thousands of generations if **M** is bound on the short term of tens to hundreds of generations to be subject to considerable sampling variance because mutations are rare events and all populations are finite? In addition, sampling effects will be compounded if mutation effects depend on genetic background and there is thus mutational “bias” due to epistastic interactions between the relevant quantitative trait loci (QTL) (Pavličev and Cheverud 2015). If mutation effects also depend on the genetic background where they first appear, then correlated selection could lead to the evolution of **M** which in turn should affect standing levels in **G** and ultimately **D** (Hermisson *et al.* 2003; Hansen *et al.* 2006; Jones *et al.* 2007, 2014).

To understand the evolution of the **G** before reaching mutation–selection balance one could start by finding out how variable is **M** between different genotypes, even if from a single population and species. As a second step, we can compare **M** with standing genetic (co)variances (**G** matrices) of populations evolving in the environment where phenotypic effects are measured. Using six independent traits of locomotion behavior as a model in the hermaphroditic nematode *Caenorhabditis elegans*, we here characterize the **M** matrix in two genotypes, using mutation accumulation (MA) experiments, and compare it with the **G** matrix of a lab-adapted population with standing genetic variation. Comparison of the two **M** matrices between founder genotypes allow us to characterize the degree of sampling and/or genetic background effects in *de novo* mutational input. In the short term of a few tens to hundreds of generations, and in the context of our particular lab environment, comparison of the **M** and **G** matrices allow us to question the relative role of pleiotropy due to mutation and LD due to selection in the maintenance of genetic (co)variances in locomotion behavior.

## Methods

### Experimental populations

To estimate **M** matrices on locomotion behavior, we employed 250 generation mutation accumulation (MA) lines from two genotypes (N2 and PB306). The details of the derivation of these MA lines can be found in Baer *et al.* (2005) and Yeh *et al.* (2017). As at each generation only one hermaphrodite was passaged by selfing, we expect that most *de novo* mutations will be fixed within each lineage unless they are extremely deleterious (Keightley and Caballero 1997; Teotónio *et al.* 2017).

We compared the **M** matrices with the **G** matrix of a lab population domesticated to standard lab conditions for 140 generations. An initial population was created from the hybridization of 16 founders (including N2 and PB306) for 33 generations that was then followed by a domestication to a defined standard lab environment for 140 generations. This standard lab environment during lab evolution is the same as that used to measure locomotion behavior at the time of reproduction in both MA lines and the lab adapted population (see below). During domestication, the population was maintained in a 4-day discrete and non-overlapping life-cycle at $N=10^{4}$ census sizes and partial-outcrossing of 60–80% (Teotónio *et al.* 2012; Mallard *et al.* 2022). The resulting population is called A6140 and contains plenty of genome-wide genetic diversity, as measured with single-nucleotide polymorphisms (Noble *et al.* 2017, 2021). From A6140, inbred lines were derived for 13–16 generations of single hermaphrodite selfing.

### Locomotion behavior

A lines were thawed from frozen stocks by blocks of ∼15 lines on 9 cm Petri dishes and grown until exhaustion of food (*Escherichia coli* HT115). This occurred 2–3 generations after thawing, after which individuals were washed from plates in M9 buffer. Adults were removed by centrifugation, and two plates per line were seeded with 1,000 larvae each. Samples were then maintained for two complete generations in a common environment characterized by a 4-day non-overlapping life-cycle with extraction of embryos from adults done by “bleaching” and developmental synchronization of L1 larvae under starvation in M9 (Stiernagle 1999). Full details about this standard lab environment used during A6140 domestication can be found in Teotónio *et al.* (2012).

Four to six generations post-thaw, adults were phenotyped for locomotion behavior at 72h post L1 larval stage seeding. At the beginning of each assay, we measured ambient temperature and humidity in the imaging room to control for their effects on locomotion. We phenotyped 54 and 62 mutation accumulation lines derived from the N2 and PB306 founders, respectively. 97 of these lines were included in two separate blocks, 1 line in 3 blocks and 18 lines were phenotyped only once. All thaw blocks contained the PB306 MA ancestor, and 15 of 16 blocks contained the N2 ancestor. For the A6140 population a total of 188 inbred lines were phenotyped, with most phenotyped twice (170), a few only once (12) and 6 lines three times, as reported in Mallard *et al.* (2022). The phenotyping of the A6140 inbred lines and of the MA lines was thus performed separately.

We imaged adults using the Multi-Worm Tracker [MWT version 1.3.0; Swierczek *et al.* (2011)] and followed the protocols detailed in Mallard *et al.* (2022). Each movie contains about 1,000 tracks of hermaphrodites (objects) with a mean duration of about 1 min. Standardized to a common frame rate (4 Hz), we filtered and extracted the number and persistence of tracked objects per movie using Choreography (Swierczek *et al.* 2011), and assigned movement states across consecutive frames as forward, still, or backward (assuming forward as the dominant direction of movement).

### Transition rates between movement states

We modeled the transition rates between forward, still and backward movement states with a continuous-time Markov process (Mallard *et al.* 2022). This process computes from our timed sequences per tracked object a set of nine transition rates between the three-movement states of still, backward, and forward. Transition rates are a measure of how fast the worms transition from each state to another one. Modeling of transition rates considers that a continuous-time Markov process is a stochastic process and assumes that the worms change movement state given a matrix **Q**. The coefficients of **Q** matrix define the probabilities of observing a worm in state *j* while knowing its previous state *i* after a waiting time Δt. Thus, the coefficients of **Q**, noted $q_{i,j}$, are the transition rates from state *i* to state *j* (off-diagonal elements being for i≠j, and with $q_{i,j}>0$). This definition constrains self-transition rates (diagonal elements) to be of the opposite sign of the sum of the two transition rates leaving a given state:


(2) $$q_{i,i}=-\sum_{\;j\neq i}q_{i,j}.$$

The probability of leaving a given movement state towards any other state is thus one minus the probability of remaining in the same state. We consider that the 6 transition rates between movement states are modeled independently and ignored self-transition rates.

Estimation of the transition rates per movie has also been detailed in Mallard *et al.* (2022). Log-likelihood models were specified in RStan (Stan Development Team (2018), R version 3.3.2, RStan version 2.15.1), which performs Bayesian inference using a Hamiltonian Monte Carlo sampling to calculate the posterior probability of the parameters given the observed data. We retained the means of the posterior distributions as the per-plate transition rates for all the subsequent analyses. Because $q_{i,j}>0$, subsequent analyses were performed on the natural log scale.

### Background effects and mutational bias

To determine the extent of genotype background differences and mutational bias between founder MA genotypes, we analyzed the six independent mean log transition rates ($q_{k}$) in the MA lines using linear mixed models:


(3) $$\ln\;(q_{k})=\alpha_{N2}+\beta_{MA}+\delta_{PB306}+\eta_{MA(PB306)}+\zeta_{lineID}+\eta_{block}+\epsilon$$

with $\alpha_{N2}$ being the mean transition rate in the founder N2 genotype before MA accumulation, $\beta_{MA}$ the mean difference between ancestral and MA lines in the N2 genotype, $\delta_{PB306}$ the mean difference between the two ancestral backgrounds and $\eta_{MA(PB306)}$ the interaction term assessing the mean difference between the N2 and PB306 MA lines. $\zeta ∼N(0,\sigma^{2})$ and $\eta ∼N(0,\sigma^{2})$ are the random effects of MA line identity and assay block, $\epsilon ∼N(0,\sigma^{2})$ is the residual error.

Differences of $\beta_{MA}$, $\delta_{PB306}$, and $\eta_{MA(PB306)}$ to zero were tested with Likelihood Ratio Tests (LRT, which is approximately $\chi^{2}$ distributed), using the *anova* command in R and as arguments the two nested models containing or not the fixed effects. As none of the six $\eta_{MA(PB306)}$ was significant, this fixed effect was removed before estimating the significance of the other two effects. We checked for homogeneity of variances between the four groups with Levene’s test on the residuals of the final model (without $\eta_{MA(PB306)}$).

### M-matrices

We estimated **M**-matrices separately for each founder genotype (N2 and PB306). The 6 transition rates $q_{k}$ were fitted as a multivariate response variable y in the model:


(4) $$\ln\;(q_{k})=\mu_{k}+\beta_{T}\;*\;T+\beta_{H}\;*\;H+\beta_{D}\;*\;D+\zeta_{lineID}+\eta_{block}+\epsilon ,$$

where $\mu_{k}$ is the general mean (intercept) of each of the $q_{k}$ traits and $\beta_{i}$ the fixed environmental effects of temperature (T), log density (D), and relative humidity (H) for trait $q_{k}$. ζ, η, and ϵ as defined above. We then estimated a matrix of genetic (co)variance as half the line covariance matrix ($\zeta_{lineID}$), as we assumed mutations in the inbred lines are homozygous.

Models were fit with the R package MCMCglmm (Hadfield 2010). We defined priors as the phenotypic variances for each trait. Model convergence was verified by visual inspection of the posterior distributions and by ensuring that chain autocorrelation remained below 0.05. We used 50,000 burn-in iterations, a thinning interval of 10 and a total of 500,000 MCMC iterations.

For each of the two **M** matrices, we constructed 1,000 randomized **M** matrices to generate a null distribution. These randomized matrices were then used for the subsequent construction of null distributions (see next section). For this, we randomly shuffled MA line and block identities and fit the above equation to obtain 1,000 posterior distributions. As discussed in Walter *et al.* (2018), the significance of the posterior mean variance-covariance estimate is based on the overlap between the posterior null distribution of the posterior mean with the observed posterior mean. Under homogeneity of variances between the groups being compared, as is the case here, differences between the estimated empirical distributions can be inferred when their 83% credible intervals do not overlap (Austin and Hux 2002).

### Comparing M matrices

To compare the **M** matrices of founder genotypes, we performed eigendecomposition of each N2 or PB306 **M** matrix independently. The resulting first eigenvectors usually contain most of the genetic variance due to mutation, the genetic variance that is measured by the eigenvalue λ, and can thus be called $m_{max}$. Random sampling expectations for the six λ, given the number of measured MA lines, were computed once by shuffling block and MA line identity per N2 or PB306 genotype. To obtain a null value along each eigentrait measuring noise, we rotated the 1,000 random matrices along the six eigentraits of the observed dataset corresponding to each genotype (see the previous section).

We then compared the relative direction of the phenotypic dimension with more genetic variance between N2 and PB306 by computing the angle between their respective $m_{max}$:


(5) $$\Theta =\frac{180}{\pi }cos^{-1}\left(\frac{PB306_{m_{max}}\cdot N2_{m_{max}}}{\left\|PB306_{m_{max}}\|*\|N2_{m_{max}}\right\|}\right).$$

As both $m_{max}$ and $-m_{max}$ are the first eigenvector of the **M** matrix and their eigenvalue $\lambda_{m_{max}}$ a single (positive) scalar, Θ values between 90° and 180° were transformed so that Θ always remains between 0° and 90° (**Θ′** = 180∘ − **Θ**, which results from using $-m_{max}$ instead of $m_{max}$ in equation (5)). The null expectation for Θ when using random vectors of a matrix with dimension two is then 45°. For matrices of higher dimension (with a higher number of traits), such as our **M** matrices, the angle will be higher than 45°, increasing asymptotically towards 90° as the number of dimensions goes to infinity (simulations not shown). Credible intervals for the null angle of Θ were obtained by sampling 1,000 pairs of random vectors from a uniform distribution $U^{6}(-1,1)$.

Additionally, we used a metric introduced by Noble *et al.* (2019) to estimate the amount of genetic variance in one M matrix along the $m_{max}$ of a second matrix. For this, we first computed the amount of genetic variance of background x along the $m_{max}$ of the background y (noted $y_{m_{max}}$):


(6) $$e_{y_{m_{max}}}^{x}=\frac{y_{m_{max}}^{T}\cdot M_{x}\cdot y_{m_{max}}}{\|y_{m_{max}}{\|}^{2}}$$

Then we defined Π as the ratio between the genetic variance along $y_{m_{max}}$ and the maximum amount of variance in any phenotypic direction in the second M matrix (noted here $\lambda_{max}^{x}$, the first eigenvalue of the **$M_{x}$** matrix):


(7) $$\Pi =\frac{e_{y_{m_{max}}}^{x}}{\lambda_{max}^{x}}$$

Π values are comprised between 0 (no genetic variance along the $m_{max}$ phenotypic dimension) and 1 (when the $m_{max}$ of the two matrices can be said to be aligned). The null expectation of Π is (Noble *et al.* 2019):


(8) $$\Pi_{0}=\frac{\bar{\lambda }}{\lambda_{max}^{x}}$$

where $\bar{\lambda }$ is the mean of all six eigenvalues of the projected $M_{x}$, the average genetic variance across any phenotypic dimensions. Credible intervals of Π and $\Pi_{0}$ were obtained by sampling 1,000 times the posterior distributions of the **M** matrices.

### Comparing M and G matrices

We compared the **M** matrices of the two inbred founders of lab evolution, N2 and PB306, with the **G** matrix of an outbred lab-adapted population A6140. **G** matrix estimation of A6140 is detailed in Mallard *et al.* (2022). The total amount of genetic (co)variances found in the N2 and PB306 **M** matrices and the A6140 **G** matrix depends on many factors, such as mutation rate and effects, which determine the sampling of a finite number of mutations, given the number of generations of MA (or equivalently number of MA lines measured) or the extent of drift and selection experienced by A6140 during its history (see Introduction). Here, we want to compare the relative amount and orientation of genetic (co)variances within and between locomotion traits. To compare **M** and **G** matrices, they thus need to be standardized on a common phenotypic scale. For this, we computed three new matrices by scaling genetic (co)variances by the total phenotypic variation and centering transition rates to a mean of zero and then dividing by the mean standard deviation among transition rates. Specifically, for each of our three groups of inbred lines:


(9) $$\ln\;(q_{k})=\frac{\ln\;(q_{k})-\mu_{k}}{\bar{\sigma }}$$

with $\mu_{k}$ being the mean transition rate values, and $\bar{\sigma }$ the mean standard deviation of the six transition rates ($\sigma_{k}$). In this manner, the mean standard deviation (${\bar{\sigma }}^{\prime }$) of our six traits is one, though each of the trait’s standard deviation ($\sigma_{k}^{\prime }$) is proportional to its initial value ($\sigma_{k}$). We then ran the same models on each of the N2, PB306, and A6140 samples independently (equation (4)). For the A6140 model, we added a fixed effect of the year of assay blocks; see Mallard *et al.* (2022) for details on how A6140 lines were phenotypes and analyzed. We verified that the total phenotypic variance assigned to random effects (genetic and residual) is similar between populations and is not affected by differences in the fixed effects variables (not shown).

To compare the size and shape of standardized **M** and **G** matrices, we performed eigentensor analysis (Hine *et al.* 2009; Aguirre *et al.* 2014). MCMCglmm was used for computation while accounting for sampling variance (Morrissey and Bonnet 2019). Eigentensors are 4-dimensional objects describing genetic variation that can be decomposed into eigenvectors maximizing in orthogonal phenotypic space the amount of genetic differentiation between the three matrices. The first of these eigenvectors, usually explaining most genetic differentiation, is called $e_{11}$. Besides estimating the amount of genetic variance in $e_{11}$ (as measured by its eigenvalue), we also compared the angle between $g_{max}$ of the A6140 with the $m_{max}$ of N2 or PB306, as above in equation (5) by replacing $m_{max}$ with $g_{max}$. $g_{max}$ is the phenotypic dimension encompassing most genetic variance in the lab-adapted population. These angles reveal if the direction of the phenotypic dimension encompassing most genetic variance is aligned between A6140 and N2 or PB306. We also projected the two **M** along $g_{max}$ (Π; equation (6)), with credible intervals being obtained from the posterior estimates of the **M** matrices.

## Results

### Mutation accumulation

**Fig. 1.**
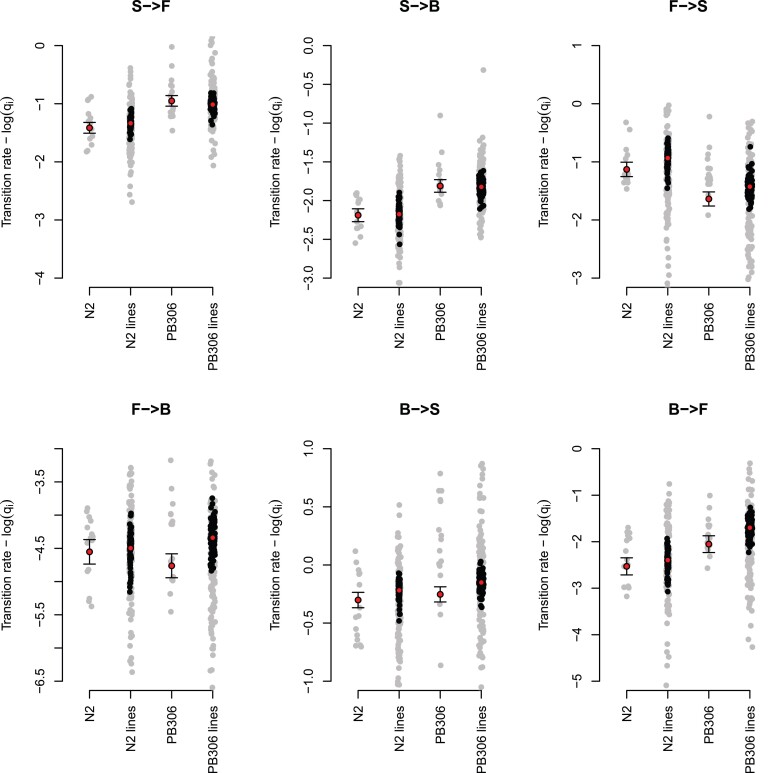
Background effects and mutational bias in locomotion behavior. Each plot shows the transition rates between backward (B), forward (F), and still (S) movement states, with left-to-right letter ordering indicating the direction of movement. Red circles show the mean for the N2 and PB306 ancestor genotypes before and after MA. Gray dots show the uncorrected measurements and black the best linear unbiased predictors of the MA line means obtained from equation (3). Error bars in the ancestor genotypes are the standard error of the mean. See Table 1 for the statistical support in genotype background effects and mutation bias.

Before MA, the two genotypes show different transition rates, with the N2 genotype being on average less active than the PB306 genotype. This is because the N2 transition rates leaving the still state are smaller than for the PB306 genotype while the forward to sill transition rate is higher (Fig. 1 and Table 1). To estimate the degree of mutation bias in locomotion behavior, we compared the mean transition rates of the ancestral genotype founders to the mean of their respective MA lines. We find that the two transition rates towards the still movement state (FS and BS) showed a significant increase from the ancestor after 250 generations of MA in both genotypes (Fig. 1 and Table 1).

**Table 1.** We tested for a mean difference between the two genetic backgrounds ($\delta_{PB306}$, equation (3)) and for mutational bias ($\beta_{MA}$, equation (3)).

Transition rate	Background		Mutational bias		Levene’s test
	Mean effect ± s.e.	*P*-value	Mean effect ± s.e.	*P*-value	*P*-value
SF	0.44±0.05	<0.0001	0.010±0.070	0.89	0.69
SB	0.37±0.04	<0.0001	0.004±0.058	0.95	0.28
FS	−0.50±0.07	<0.0001	0.188±0.089	0.034	0.47
FB	0.16±0.10	0.11	0.199±0.138	0.15	0.15
BS	0.05±0.03	0.12	0.094±0.045	0.038	0.31
BF	0.51±0.10	<0.0001	0.221±0.135	0.099	0.22

We report the mean difference and estimated standard error for each test. *P*-values were obtained after an LRT following a $\chi_{1}^{2}$ distribution. We also report the P-values of Levene’s test, modeling the homogeneity of variances between the four groups being compared (two founder ancestor genotypes and respective MA lines).

**Fig. 2.**
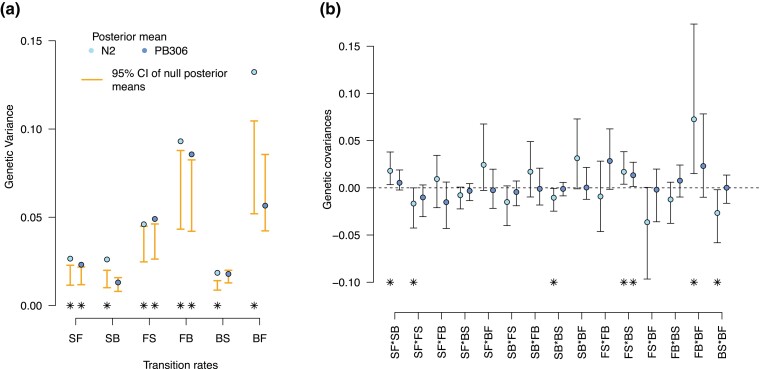
**M**-matrices for N2 and PB306 genotypes. a) Genetic variance estimates for each transition rate. Lettering indicates backward (B), forward (F) and still (S) movement states. The posterior means are compared to the 95% CI of the randomized null M matrices (orange bars). We detect significant genetic variance for all traits in the N2 background and for 3 out of 6 traits in the PB306 background (stars indicating significance at α<0.05). b) Genetic covariances estimates between transition rates. (Co)variances estimates are non-null if the 95% CI of the distribution does not overlap zero (stars indicating significance at α<0.05). Mean and CI interval values of genetic (co)variances can be found in Supplementary Table S1 as well as the 95% CI of the null distributions.

We next estimated the N2 and PB306 **M** matrices and found that most of the transition rate genetic variances do not overlap the randomized null 95% credible intervals (Fig. 2 and Supplementary Table S1): all of the N2 genotype estimates and three out of six estimates from the PB306 genotype are not expected from random sampling alone. For genetic covariances between transition rates, all estimates but one of PB306 are not different from zero. In N2, 6 covariances are different from zero.

**Fig. 3.**
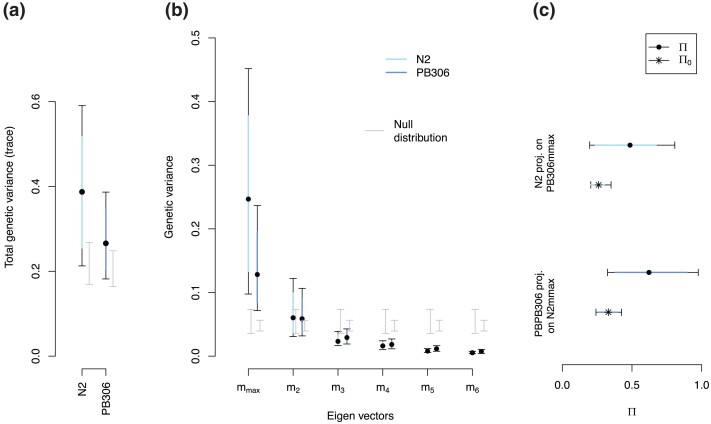
**M** matrix comparison between N2 and PB306. a) Shown is the total amount of genetic variance as measured by the trace of the **M** matrices. For both N2 and PB306, the total genetic variance is different from the randomized null distribution (gray, mean ±95% CI), but there is no difference between N2 and PB306 (colored, 83% CI). b) Spectral decomposition of the **M**-matrices indicate that the phenotypic dimension encompassing most genetic variance (measured by the λ eigenvalue of the first eigenvector $m_{max}$), is different from the null distribution for both N2 and PB306 (gray, mean ±95% CI), but N2 and PB306 do not differ in this $m_{max}$ dimension (colored, 83% CI). **C.** The normalized projection of the **M** matrices on the other background’s $m_{max}$ axis ranges from 0 to 1 (with 1 meaning perfect alignment, see Methods). For each matrix from our posterior distribution, we compute Π (dots and 83% and 95% bars; see equation (6)) and the null expectation ($\Pi_{0}$, 83%, and 95% bars with star; see equation (7)). For each background, there is more variance projected than under a null expectation because the mean estimates do not overlap the 95% null CI (Supplementary Table S2).

The total amount of genetic variance for locomotion behavior does not differ between the N2 and PB306 genotypes (Fig. 3a). Eigen decomposition of the two **M** matrices further reveals no differences between genotypes along the phenotypic dimension encompassing most genetic variation, the first eigenvector $m_{max}$ (Fig. 3b and Supplementary Table S2). Both **M** matrices have high uncertainty and calculating the angle between their respective $m_{max}$ is uninformative (Supplementary Fig. S1). On the other hand, we observe that there is more mutational variance of the first genotype along the major axis of genetic variation of the second genotype than under a null hypothesis based on sampling a limited number of lines (Fig. 3c and Supplementary Table S3). This latter result also shows an overall congruence between the two matrices.

### Standing and mutation genetic variation

**Fig. 4.**
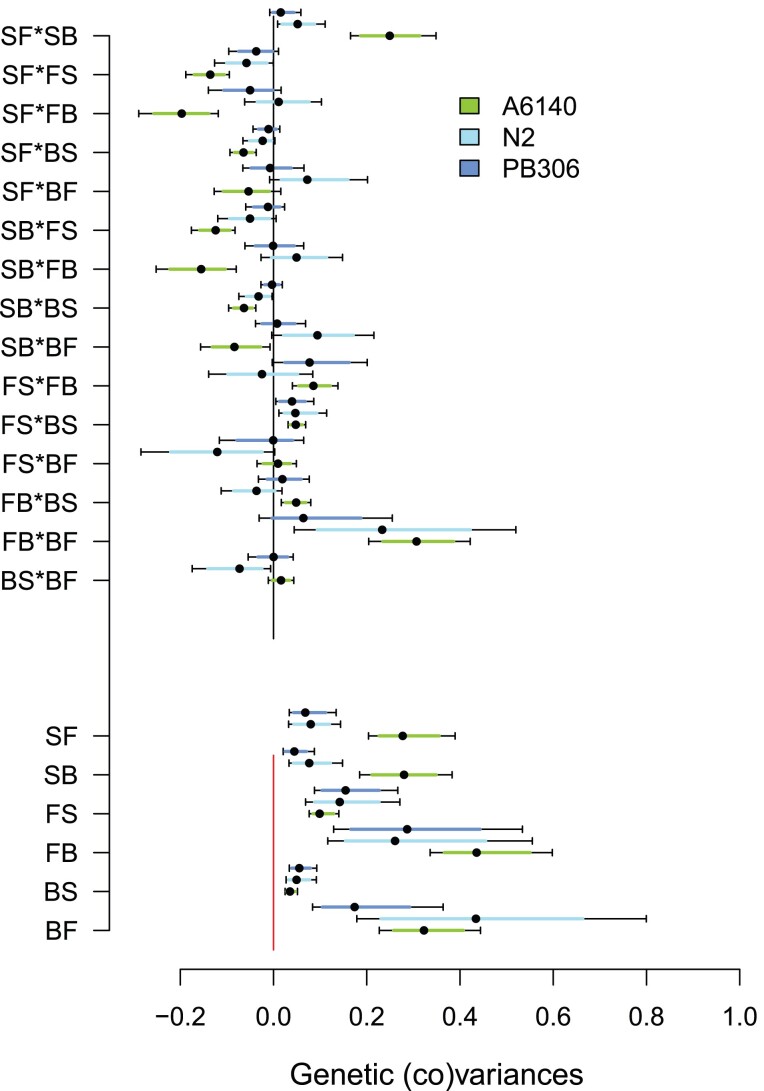
Standing and mutation genetic (co)variances for locomotion behavior. Lettering indicates backward (B), forward (F), and still (S) movement states, left to right indicating the direction of movement. The bottom six entries are the diagonal genetic variance estimates for each transition rate, while top 15 entries the off-diagonal genetic covariances estimates between transition rates. Green for the lab-adapted population (A6140), cyan for the N2 genotype, blue for the PB306 genotype. Dots show the mean of posterior distribution with bars being the 95% credible intervals. Distributions can be differentiated whenever their 83% credible intervals do not overlap (colored bars). All estimates are standardized by on a common scale by dividing each genetic (co)variance by the total phenotypic variance in each population (equation (9)).

We compared the size and orientation of the N2 and PB306 **M** matrices with the **G** matrix of an outbred lab-domesticated population (A6140) containing standing genetic variation (see Introduction and Methods). Detailed characterization of this **G** matrix has been presented in Mallard *et al.* (2022). All 6 locomotion traits showed significant genetic variance in this population (Fig. 4). The eigendecomposition of the matrix showed an excess of variance compared with the randomized matrix in the first three eigentraits, encompassing more than 70% of the total genetic variance (Supplementary Table S4).

To compare the two **M** matrices of N2 and PB306 with the **G** matrix, they were standardized to a common phenotypic scale (see Methods). We find that leaving the still movement state (SF and SB transition rates) contains more standing than mutational genetic variances (Fig. 4), and that, correspondingly, the genetic covariances between these two transition rates with each other and with other transition rates differ between the lab-domesticated population and the N2 and PB306 genotypes.

**Fig. 5.**
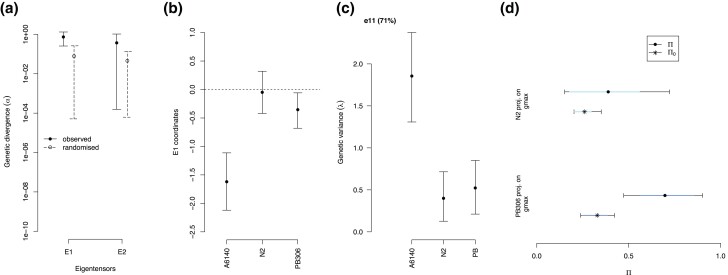
Standing versus mutation genetic (co)variances for locomotion behavior. a) Eigentensor decomposition of the 3 matrices, 2 **M** matrices from the N2 and the PB306 genotypes, and the **G** matrix from the A6140 lab-adapted population (from Fig. 4). The genetic variance explaining differences between matrices are shown as the mode and 95% credible interval of the posterior distributions of the first ($E_{1}$) and second ($E_{2}$) eigentensors, along with the expected distributions by sampling alone (line and dashed, respectively). b) In the first eigentensor ($E_{1}$), the coordinates of the three matrices. The lab-adapted population has the largest absolute values, which drives most of the differentiation seen in panel A. c) The first eigenvector ($e_{11}$) of the first eigentensor ($E_{1}$) is the one where most genetic differences between the lab-adapted population and the N2 and PB306 genotypes are found (71% of the variance found in E1). d) The normalized projection of the **M**-matrices on the $g_{max}$ of the domesticated population (dots and 83% and 95% colored bars). Here only the PB306 **M**-matrix has more genetic variance along $g_{max}$ than under a null expectation ($\Pi_{0}$, asterisks, and bars; see equation (7)). Moreover, the N2 genotype is not more aligned with the **G** matrix than expected by chance and has significantly less aligned variance than the PB306 genotype (using the 83% CI criteria; Supplementary Table S3). The angles between the two $m_{max}$ and $g_{max}$ show the same trend between the two backgrounds (Supplementary Fig. S2).

Tensor analysis of the three genetic (co)variance matrices reveals that only the first eigentensor is different from random expectations (Fig. 5a and Supplementary Table S5), and that most genetic differentiation is due to the lab-adapted population (Fig. 5b). Further decomposition of this eigentensor $E_{1}$ indicates that the lab-adapted population is genetically differentiated from the N2 and PB306 genotypes only in the first eigenvector $e_{11}$ (Fig. 5c). The angle between the A6140 $g_{max}$ and the PB306 $m_{max}$ shows differentiation, even if the uncertainty remains high because of low confidence in the genetic structure of the **M** matrices (Supplementary Fig. S2). We also observed a difference between our two **M** matrices when projecting their variance along $g_{max}$ (Fig. 5d and Supplementary Table S6): PB306 shows an excess of mutational variance which is aligned with standing genetic variation when compared with the mutational variance of the N2 genotype.

## Discussion

Showing if and how the **M** matrix differs among genotypes within species is important to understand short- and long-term phenotypic evolution (see Introduction). Here, we have described the **M** matrix for six traits in locomotion behavior in two genotypes of *C. elegans*, after MA in a nearly neutral manner for 250 generations (Baer *et al.* 2005; Yeh *et al.* 2017). We also compared the two **M** matrices with the **G** matrix of a population domesticated in the lab for 140 generations (Noble *et al.* 2017; Mallard *et al.* 2022), a lab-adapted population ultimately derived from the hybridization of 16 founder genotypes (Teotónio *et al.* 2012), including the two genotypes phenotyped for **M**.

Before MA, the two genotypes, N2 and PB306, show different locomotion behaviors, with PB306 being more active than the N2 genotype (Fig. 1 and Table 1). This result is unsurprising as the N2 strain was domesticated for a long period to laboratory conditions similar to ours, where movement is not necessarily favored and what would be deleterious mutations in the wild have fixed (Sterken *et al.* 2015). We further find that mutational bias for the transition rates between forward and still movement states, and between backward and still movement states, differs between the two genotypes (Table 1). In the N2 genotype, an increased number of mutations with time appear to have a relatively larger effect, leading to disproportionately more immobile worms, when compared with the PB306 genotype. Mutational bias occurs when the distribution of phenotypic effects depends on genetic background and thus when there are non-additive and epistatic effects between the *de novo* MA and the fixed genetic background (Halligan and Keightley 2009; Jones *et al.* 2014; Saxena *et al.* 2018; Schweizer and Wagner 2020). In our case, however, one should be cautious in interpreting the presence of epistasis because the phenotypic effect distribution of new mutations was barely sampled (Keightley *et al.* 2000; Peters and Keightley 2000; Jasmin and Lenormand 2016). Given the number of generations in any MA experiment and the small number of lines assayed here, each MA line carries an idiosyncratic number of mutations that do not comprehensibly target the QTL underlying presumed polygenic traits such as locomotion behavioral traits.

There are no detectable differences in size or orientation between the two **M** matrices (Figs. 2, 3; Supplementary Fig. S1, Tables S2 and S3). For **M** matrix size, summarized in the amount of genetic variation in locomotion behavior (Fig. 3a), our result is consistent with the observation that N2 and PB306 genotypes show similar mutation rates and molecular spectra for single nucleotide polymorphisms (Denver *et al.* 2012; Rajaei *et al.* 2021). However, a more recent study with an extensive set of MA lines from both genotypes has indicated that mutation rates for short-tandem repeats (STRs) were different (Zhang *et al.* 2022). Given that some of these STRs have also been shown to affect QTL for polygenic traits (Zhang *et al.* 2022), one could have expected that the two **M** matrices for locomotion behavior would be different. Regarding **M** matrix orientation, it should depend on pleiotropy and therefore on the amount of genetic covariances between transition rates (Lande 1980; Phillips and McGuigan 2006). We find that for both genotypes genetic covariances between transition rates are small and mostly indistinguishable from zero (Fig. 2b and Supplementary Table S1). So, it is not surprising that the two genotypes do not differ in **M** matrix orientation. Consistent with this result, it has been shown that when significant, mutational correlations between vulval and fitness-related traits also appear to be similar in sign and magnitude among the two genotypes used here (Braendle *et al.* 2010). However, there is high uncertainty in estimating genetic covariances because they cannot be larger than genetic variances of the transition rates, and only a few MA lines were phenotyped. The fact that one phenotypic dimension of the eigendecomposition analysis encompasses most of the mutational variance ($m_{max}$) and align well between genotypes (Fig. 3b,c; Supplementary Tables S2 and S3), suggests that traits share genetic variation, and hence that pleiotropy was similarly extensive between genotypes (Walsh and Blows 2009).

In line with selection impacting the evolution of the **G** matrix (see Introduction), the **G** matrix of the lab-adapted population was different from the **M** matrices of the two genotypes. The two kinds of **M** and **G** matrices are different due not only to more genetic (co)variances in the lab population between traits related to worm activity and leaving the still state but also because there was relatively less genetic variance created by mutation in leaving the still state than present in the lab-adapted population (Fig. 4). Differentiation between the **M** and **G** matrices is driven by the lab-adapted population (Fig. 5b,c and Supplementary Table S5), with no differences being found between the two **M** matrices. The exception to this result is the projection of the **M** matrices through the phenotypic dimension of maximal standing genetic variation ($g_{max}$), indicating that the PB306 genotype has more than expected genetic variance along this dimension (Fig. 5d and Supplementary Table S6). The angle between the PB306 $m_{max}$ and the lab-adapted population $g_{max}$ is also smaller than expected by chance (Supplementary Fig. S2). In other words, the N2 **M** matrix might be more different in orientation from the **G** matrix of the lab-adapted population than the PB306 **M** matrix, despite no differences being found when they are directly compared with each other (Figs. 2 and 3; Supplementary Fig. S1).

A relative change of standing genetic variances to mutational variances could either be interpreted to reflect the selective purging of deleterious mutations in the lab or the selective maintenance of variation in some traits above that expected with neutrality. *Caenorhabditis elegans* populations in nature are found highly inbred and isogenic, due to a long history of predominant selfing, extensive selective sweeps, and background selection (Cutter 2006; Rockman *et al.* 2010; Andersen *et al.* 2012). Hybridization of natural isolates leads to outbreeding depression (Dolgin *et al.* 2007; Chelo *et al.* 2013), in part due to the disruption of gene complexes (Seidel *et al.* 2008; Gaertner *et al.* 2012). With a haploid base substitution mutation rate of $2.5\times 10^{9}$ (Saxena *et al.* 2018) and effective population size of $10^{3}$ during lab evolution (Chelo and Teotónio 2013), most of the single nucleotide polymorphism mutations that we previously found segregating in the lab population should not have been observed (Noble *et al.* 2017), assuming that most are deleterious and partially dominant. We have further shown that lab adaptation not only involved maintenance of excess heterozygosity, due to overdominant loci interacting in a non-additive fashion (Chelo and Teotónio 2013), but also that LD, though much reduced from that found among founders, was still important for several genomic regions potentially encompassing many QTL (Noble *et al.* 2017, 2021). Among these genomic regions, LD was also higher than expected, probably because of additive-by-additive epistasis between QTL (Noble *et al.* 2017). Taken together, these observations suggest that the hybridization of the 16 founders created the opportunity for the maintenance of LD by selection, in turn explaining why higher levels of genetic (co)variances in transition rates from a still movement state are measured in the lab population than expected with mutation alone.

How do our results compare with other studies, in other organisms and with different kinds of traits? To our knowledge, there has been only one other study in which the **M** matrix has been characterized in two genotypes of the same species. Using an MA design, Houle and Fierst (2013) compared **M** matrices for a set of up to 18 traits related to wing morphology between two *Drosophila melanogaster* genotypes, genotypes that were known to have different mutation rates and molecular spectra (Schrider *et al.* 2013). When considering that alleles fixed within each MA line have additive effects, Houle and Fierst (2013) found that the two **M** matrices differ in size and orientation. Interestingly, when considering the heterozygous effects in crosses between MA lines, the two **M** matrices continued to differ but were dissimilar from additive **M** matrices. Directional dominance and/or epistasis was not detected, however, indicating that MA experiments at small population sizes are adequate, as a first approximation, to estimate the **M** matrix in organisms such as Drosophila spp. where inbreeding depression effects are important. In a later study, Houle *et al.* (2017) compared an “averaged” **M** matrix between the two genotypes with the orientation of the **G** matrix from a *D. melanogaster* population and with the divergence **D** matrix from many Drosophilid species spanning 40 million years, having found that they were all congruent in orientation with each other, if not in size. The authors concluded that mutation predicts standing genetic variation and long-term phenotypic divergence. However, because only a single **M** matrix was used for prediction, it is unclear whether the **M** matrix can evolve to match the orientation of the selection surface as expected from simulation studies (Jones *et al.* 2007, 2014).

Dugand *et al.* (2021) have estimated a single **M** matrix for five wing morphological traits but in the context of an evolving *D. serrata* outbred population for 14 generations, and from which a **G** matrix could be simultaneously estimated using a defined pedigreed experimental design (McGuigan *et al.* 2015). With this design, mutations appear, segregate, and are fixed among many genotypes during evolution, and dominance and epistatic effects are explicitly accounted for. **M** and **G** are further estimated on a common phenotypic scale, as there are no differential environmental effects, allowing then for the inference of selection in the long term of mutation-selection balance (Sztepanacz and Blows 2017). Dugand *et al.* (2021) found that **M** and **G** were congruent for most phenotypic dimensions where there was standing genetic variation, except in one phenotypic dimension where less standing genetic variation was found than that expected for neutral traits with significant mutational variance. Not surprisingly, for such a short-term experiment, selection on standing genetic variation seemed more important than mutation.

In conclusion, few studies have attempted to measure how variation in mutational (co)variances determines standing genetic (co)variances and, eventually, the phenotypic divergence between populations. While the study of Dugand *et al.* (2021) is outstanding in the excellence of design and analyses, it is unlikely to be much replicated because experiments are prohibitively expensive. MA experiments have interpretation problems, but they will be in a less expensive position to estimate **M** from specific genotypes. Given that these rare studies cannot be compared due to differences in design, analyses, and statistical power, little can be said about **M** matrix differences between genotypes, and if so why, or how the **M** matrix evolves, if it does, or about predicting phenotypic divergence from mutation in the short or long term. For locomotion behavior in the predominantly selfing *C. elegans*, hybridization of extant genotypes restructures genetic covariances so that selection in the short-term at new phenotypic dimensions is possible when mutation has little influence.

## Supplementary Material

jkac335_Supplementary_Data

## Data Availability

Data for the domesticated lab population (A6140) has been published in Mallard *et al.* (2022). All new data, sample sizes, modeling results, and R code can be found in https://github.com/ExpEvolWormLab/Mallard˙Mutation. Supplementary material are available at *GENETICS* online.

## Notes

*Communicating editor: D.-J. de Koning*
