# Supplementary material for: Variation in mutational (co)variances
Source: G3 (Bethesda). 2022 Dec 22;13(2):jkac335. doi: 10.1093/g3journal/jkac335 (PMC9911065; doi:10.1093/g3journal/jkac335)
Supplement: jkac335_Supplementary_Data [file jkac335_supplementary_data.pdf]

Supplementary figures and tables for: variation in mutational (co)variances

François Mallard[1*]

Luke Noble[1]

Charles F. Baer[2]

Henrique Teotónio[1,*]

1. Institut de Biologie de l'École Normale Supérieure, CNRS UMR 8197, Inserm U1024, PSL Research University, F-75005 Paris, France;

2. Department of Biology, University of Florida Genetics Institute, University of Florida, Gainsville, Florida 32611, U.S.A.;

∗ Corresponding authors: mallard@bio.ens.psl.eu, teotonio@bio.ens.psl.eu.

1 Supplementary Figures

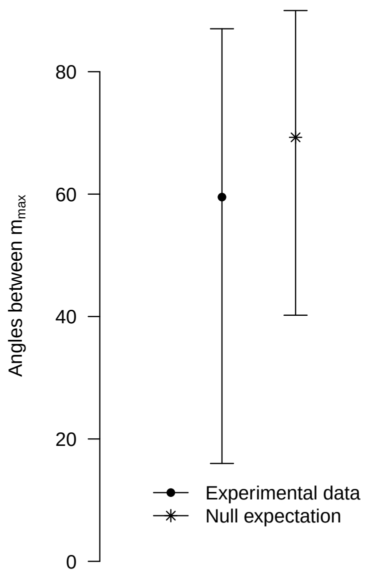

Figure S1: Angle between the m_{max} of the N2 and PB306 M matrices (from Figure 3B, see **??**). The error bars show the 95% CI. There is a large uncertainty in this metric and it does not differ from the null expectation (star and bar; see Methods).

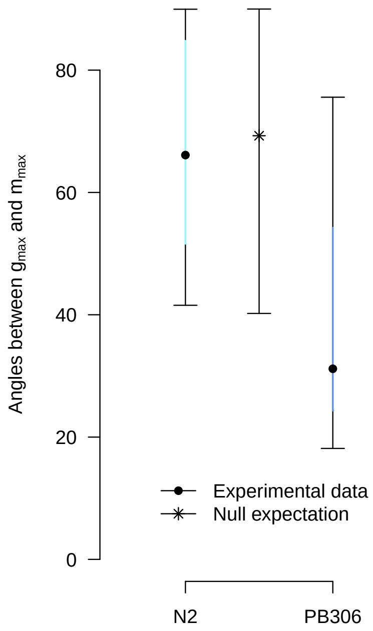

Figure S2: Dots with 83% and 95% colored bars show the angle between the g_{max} of the lab-adapted population A6140 with the m_{max} of the N2 and PB306 (from Figure 3B; equation **??**). For the PB306 genotype, the posterior mean is lower than the 95% CI of the null expectation (middle bar with star; see Methods).

2 Supplementary Tables

N2 genotype

	SF	SB	FS	FB	BS	BF
**SF**	**0.0265 [0.011;0.0485]**	0.018 [0.0035;0.038]	-0.0165 [-0.0425;0]	0.0095 [-0.021;0.0345]	-0.008 [-0.0225;0.001]	0.0245 [-0.0025;0.0675]
**SB**	0.018 [0.0035;0.038]	**0.026 [0.011;0.05]**	-0.015 [-0.04;0.002]	0.017 [-0.0095;0.049]	-0.0105 [-0.0245;-5e-04]	0.0315 [-0.001;0.073]
**FS**	-0.0165 [-0.0425;0]	-0.015 [-0.04;0.002]	**0.046 [0.024;0.0915]**	-0.009 [-0.0465;0.0285]	0.017 [0.004;0.0385]	-0.0365 [-0.0965;5e-04]
**FB**	0.0095 [-0.021;0.0345]	0.017 [-0.0095;0.049]	-0.009 [-0.0465;0.0285]	**0.093 [0.0395;0.1855]**	-0.0125 [-0.0375;0.006]	0.0725 [0.015;0.1735]
**BS**	-0.008 [-0.0225;0.001]	-0.0105 [-0.0245;-5e-04]	0.017 [0.004;0.0385]	-0.0125 [-0.0375;0.006]	**0.0185 [0.009;0.031]**	-0.0265 [-0.058;-0.002]
**BF**	0.0245 [-0.0025;0.0675]	0.0315 [-0.001;0.073]	-0.0365 [-0.0965;5e-04]	0.0725 [0.015;0.1735]	-0.0265 [-0.058;-0.002]	**0.1325 [0.058;0.2675]**

PB306 genotype

	SF	SB	FS	FB	BS	BF
**SF**	0.023 [0.011;0.0425]	0.0055 [-0.0025;0.019]	-0.01 [-0.0305;0.003]	-0.015 [-0.043;0.006]	-0.003 [-0.0135;0.0045]	-0.0025 [-0.022;0.02]
**SB**	0.0055 [-0.0025;0.019]	0.013 [0.0065;0.0275]	-0.0045 [-0.019;0.007]	-0.001 [-0.018;0.021]	-0.001 [-0.0085;0.0055]	5e-04 [-0.012;0.0215]
**FS**	-0.01 [-0.0305;0.003]	-0.0045 [-0.019;0.007]	0.049 [0.028;0.0845]	0.0285 [-0.0015;0.0625]	0.0135 [0.0015;0.027]	-0.002 [-0.036;0.02]
**FB**	-0.015 [-0.043;0.006]	-0.001 [-0.018;0.021]	0.0285 [-0.0015;0.0625]	0.0855 [0.043;0.1695]	0.0075 [-0.0095;0.024]	0.023 [-0.01;0.0785]
**BS**	-0.003 [-0.0135;0.0045]	-0.001 [-0.0085;0.0055]	0.0135 [0.0015;0.027]	0.0075 [-0.0095;0.024]	0.018 [0.0105;0.029]	0 [-0.0165;0.0135]
**BF**	-0.0025 [-0.022;0.02]	5e-04 [-0.012;0.0215]	-0.002 [-0.036;0.02]	0.023 [-0.01;0.0785]	0 [-0.0165;0.0135]	0.0565 [0.0275;0.115]

95% CI of the null variance estimates (1000 randomized matrices)

	SF	SB	FS	FB	BS	BF
**N2**	[0.0115 - 0.0228]	[0.0101 - 0.0199]	[0.0247 - 0.0449]	[0.0433 - 0.0878]	[0.0087 - 0.0141]	[0.052 - 0.1046]
**PB306**	[0.0118 - 0.0218]	[0.008 - 0.0158]	[0.0263 - 0.0462]	[0.0421 - 0.0825]	[0.0128 - 0.02]	[0.0423 - 0.0856]

Table S1: **M** matrices of the N2 (top) and PB306 (bottom) genotypes, with 95% credible intervals. Diagonal entries show genetic variances, while off-diagonal entries genetic covariances (the matrices are symmetric).

	N2							PB306					
	m_{max}	m_2	m_3	m_4	m_5	m_6		m_{max}	m_2	m_3	m_4	m_5	m_6
Eigenvalues	0.4310	0.1233	0.0523	0.0437	0.0210	0.0138		0.2279	0.1163	0.0587	0.0419	0.0256	0.0203
HPD lower	0.1952	0.0616	0.0332	0.0206	0.0114	0.0076		0.1435	0.0636	0.0382	0.0230	0.0153	0.0086
HPD upper	0.9036	0.2443	0.0779	0.0482	0.0237	0.0154		0.4731	0.2129	0.0856	0.0540	0.0327	0.0213
Proportion	0.6291	0.1800	0.0763	0.0638	0.0306	0.0201		0.4644	0.2371	0.1196	0.0855	0.0522	0.0413
Trait loadings:													
SF	-0.1715	0.3263	0.4841	0.4258	-0.3650	0.5614		-0.1988	-0.1218	-0.0688	0.8611	-0.1290	0.4276
SB	-0.2162	0.2306	0.4243	0.4156	0.3683	-0.6417		-0.0374	-0.0784	-0.1520	0.4262	0.1717	-0.8708
FS	0.2427	-0.5944	-0.1424	0.6770	0.2630	0.1999		0.4090	0.5518	0.5576	0.2242	-0.3871	-0.1312
FB	-0.5229	-0.6613	0.4513	-0.2881	-0.0448	0.0247		0.8184	-0.0216	-0.5523	0.0909	0.0511	0.1178
BS	0.1733	-0.2085	-0.0276	0.2049	-0.8070	-0.4821		0.1290	0.1881	0.3236	0.1350	0.8935	0.1633
BF	-0.7493	0.0795	-0.6007	0.2503	-0.0929	-0.0074		0.3246	-0.7992	0.5015	0.0111	-0.0555	-0.0350

Table S2: Eigendecomposition of **M** matrices for each genotype. Eigenvalues are reported with their 95% CI and the proportion of total genetic variance.

	Projection on mmax			Projection on mmax (NULL)		
	mean	95% CI	83%CI	mean	95% CI	83%CI
N2	0.49	[0.24 - 0.83]	[0.26 - 0.67]	0.26	[0.20 - 0.33]	[0.21 - 0.30]
PB306	0.61	[0.29 - 0.95]	[0.36 - 0.88]	0.33	[0.24 - 0.43]	[0.14 - 0.68]

Table S3: Projection of each **M** matrix on the other genotype m_{max} with mean, 95%CI and 83% CI

	A6140 (scaled)					
	g_{max}	g_2	g_3	g_4	g_5	g_6
Eigenvalues	1.8542	0.7990	0.1575	0.0680	0.0168	0.0036
HPD lower	1.4177	0.5173	0.1109	0.0475	0.0251	0.0147
HPD upper	2.5055	1.1039	0.2119	0.1028	0.0400	0.0255
Proportion	0.6396	0.2756	0.0543	0.0235	0.0058	0.0013
Trait loadings:						
SF	-0.4423	0.4649	0.2072	-0.3922	0.0967	0.6182
SB	-0.4261	0.4526	-0.6092	-0.0734	-0.0044	-0.4869
FS	0.2122	-0.2931	-0.2271	-0.6823	0.5883	-0.0765
FB	0.6225	0.3673	-0.5228	0.1280	-0.0387	0.4317
BS	0.1151	-0.1243	-0.0653	-0.5728	-0.7983	-0.0409
BF	0.4207	0.5855	0.5067	-0.1754	0.0760	-0.4323

Table S4: Eigendecomposition of the scaled **G** matrix of the A6140 population. Eigenvalues are reported with their 95% CI and the proportion of total genetic variance.

Eigentensor	Proportion	Eigenvalue of S	Eigenvector of eigentensor	λ	SF	SB	FS	FB	BS	BF
E1	0.76	0.74 [0.26 - 1.32]	$e_{1,1}$	-0.9621	-0.5362	-0.5537	0.2097	0.4969	0.1371	0.3101
			$e_{1,2}$	0.2570	-0.0414	-0.1734	0.5406	-0.0902	0.3292	-0.7480
			$e_{1,3}$	0.0771	-0.2289	-0.4002	-0.7402	-0.2924	0.2868	-0.2680
			$e_{1,4}$	0.0470	0.1830	-0.1030	-0.2718	0.6344	-0.5000	-0.4792
			$e_{1,5}$	-0.0120	0.7872	-0.4279	0.0005	0.1456	0.3685	0.2006
			$e_{1,6}$	0.0026	0.0719	-0.5563	0.2051	-0.4856	-0.6363	0.0517
E2	0.29	0.22 [0 - 1.04]	$e_{2,1}$	-0.9643	-0.2055	-0.2105	0.2715	-0.4867	0.1789	-0.7556
			$e_{2,2}$	-0.1884	-0.5386	-0.7501	0.1021	0.2386	0.1037	0.2630
			$e_{2,3}$	0.1758	-0.0221	-0.2214	-0.6054	-0.6981	-0.1716	0.2592
			$e_{2,4}$	0.0475	-0.1718	0.3010	0.4425	-0.4107	0.5085	0.5068
			$e_{2,5}$	0.0351	0.1741	-0.1527	0.5875	-0.2229	-0.7207	0.1793
			$e_{2,6}$	0.0130	0.7793	-0.4797	0.0913	-0.0240	0.3871	0.0616

Table S5: Eigentensor analysis of the difference between **G** and **M** matrices. The eigenvalue of each eigentensor is the amount of total difference between the matrices captured by each eigentensor. It is also shown the decomposition of each eigentensor with their trait loadings and eigenvalues (λ).

	Projection on gmax			Projection on gmax (NULL)		
	mean	95% CI	83%CI	mean	95% CI	83%CI
N2	0.38	[0.14 - 0.68]	[0.16 - 0.55]	0.26	[0.20 - 0.33]	[0.21 - 0.30]
PB306	0.69	[0.44 - 0.88]	[0.56 - 0.85]	0.33	[0.25 - 0.43]	[0.26 - 0.40]

Table S6: Projection of the two **M** matrices on g_{max} with mean, 95% CI and 83% CI (see Figure 5D).
